# Phenotypic and functional characterization of macrophages with therapeutic potential generated from human cirrhotic monocytes in a cohort study

**DOI:** 10.1016/j.jcyt.2015.07.016

**Published:** 2015-11

**Authors:** Joanna K. Moore, Alison C. Mackinnon, Dvina Wojtacha, Caroline Pope, Alasdair R. Fraser, Paul Burgoyne, Laura Bailey, Chloe Pass, Anne Atkinson, Neil W.A. Mcgowan, Lynn Manson, Mark L. Turner, John D.M. Campbell, Stuart J. Forbes

**Affiliations:** 1 MRC Centre for Regenerative Medicine, Max Born Crescent, University of Edinburgh, Edinburgh, United Kingdom; 2 Scottish Universities Life Sciences Alliance (SULSA), Max Born Crescent, University of Edinburgh, Edinburgh, United Kingdom; 3 Research, Development and Innovation, Scottish National Blood Transfusion Service, Ellen's Glen Road, Edinburgh, United Kingdom; 4 Scottish National Blood Transfusion Service, Edinburgh Royal Infirmary, United Kingdom

## Abstract

**Background aims:**

Macrophages have complex roles in the liver. The aim of this study was to compare profiles of human monocyte-derived macrophages between controls and cirrhotic patients, to determine whether chronic inflammation affects precursor number or the phenotype, with the eventual aim to develop a cell therapy for cirrhosis.

**Methods:**

Infusion of human macrophages in a murine liver fibrosis model demonstrated a decrease in markers of liver injury (alanine transaminase, bilirubin, aspartate transaminase) and fibrosis (transforming growth factor-β, α-smooth muscle actin, phosphatidylserine receptor) and an increase in markers of liver regeneration (matrix metalloproteinases [MMP]-9, MMP-12 and TNF-related weak inducer of apoptosis). CD14+ monocytes were then isolated from controls. Monocytes were matured into macrophages for 7 days using a Good Manufacturing Practice–compatible technique.

**Results:**

There was no significant difference between the mean number of CD14+ monocytes isolated from cirrhotic patients (n = 9) and controls (n = 10); 2.8 ± SEM 0.54 × 10^8^ and 2.5 ± 0.56 × 10^8^, respectively. The mean yield of mature macrophages cultured was also not significantly different between cirrhotic patients and controls (0.9 × 10^8^ ± 0.38 × 10^8^, with more than 90% viability and 0.65 × 10^8^ ± 0.16 × 10^8^, respectively. Maturation to macrophages resulted in up-regulation of a number of genes (MMP-9, CCL2, interleukin [IL]-10 and TNF-related weak inducer of apoptosis). A cytokine and chemokine polymerase chain reaction array, comparing the control and cirrhotic macrophages, revealed no statistically significant differences.

**Conclusions:**

Macrophages can be differentiated from cirrhotic patients' apheresis-derived CD14 monocytes and develop the same pro-resolution phenotype as control macrophages, indicating their suitability for clinical therapy.

## Introduction

Macrophages are a heterogeneous population of cells with diverse roles within the liver, including phagocytosis, maintaining immune tolerance and both promotion and resolution of inflammation and fibrosis through activation of hepatic stellate cells/production of cytokines and degradation of the extracellular matrix, respectively [1–4]. They are also involved in the livers' regenerative response after injury [5,6].

Hepatic macrophages can arise either from bone marrow (BM)-derived monocytes or from self-renewing endogenous cells in the liver, termed Kupffer cells [7]. Although an oversimplification given their complex function, two distinct *in vitro* macrophage phenotypes have been described; M1 (pro-inflammatory) and M2 (anti-inflammatory) [8]. M1s are associated with Th-1 CD4 T cells and induced by interleukin (IL-12, interferon-γ and lipopolysaccharide (LPS) in response to liver injury. M2s are linked to Th-2 CD4 T cells and controlled by IL-4 and IL-13 [9]. There is currently widespread use of at least four definitions of macrophage activation (M1/M2, alternative/classic activation, “regulatory” macrophages and subdivisions), and there is a requirement for common terminology and consistent use of markers across the literature [10].

Macrophages can both promote fibrogenesis by activating the pro-fibrotic cytokine transforming growth factor (TGF)-β [11] and by stimulating myofibroblast proliferation by platelet-derived growth factor (PDGF), IL-1β and tumor necrosis factor-α [12]. They are also critical for fibrosis resolution because they provide a rich source of the scar-degrading matrix metalloproteinases (MMPs) [13]. They produce factors such as MMP-9, which promote hepatic stellate cell apoptosis, needed for scar resolution [14]. They also phagocytose cellular debris, which removes potential pro-inflammatory signals [3].

It is known that there are more circulating monocytes in patients with chronic liver disease than in healthy controls, and there is a close association with this and disease progression [15]. There is also T-cell activation in cirrhosis accompanied by an increase in circulating anti-inflammatory and pro-inflammatory cytokines but attenuated cytokine production in T cells [15,16].

Previous work by our group demonstrated that administration of mature murine macrophages (and not undifferentiated monocytes) into a CCl_4_-induced murine liver injury model results in early chemokine up-regulation, leading to hepatic recruitment of endogenous macrophages, an increase in anti-inflammatory cytokines, decrease in hepatic myofibroblasts and overall improvement in clinically relevant parameters such as albumin [17].

Further work by our group [3] identified a “restorative macrophage” in the mouse that undergoes a functional switch during liver injury from an inflammatory Ly-6C^hi^ monocyte/macrophage subset to a Ly-6C^lo^ subset that is capable of resolving fibrosis. Of note, continued infiltration by Ly-6C^hi^ macrophages after the cessation of injury inhibited fibrosis regression, postulating that endogenous macrophages aid fibrosis resolution and infiltrating macrophages worsen it.

Published human data in patients with cirrhosis is lacking. It is known that endogenous macrophages are significantly increased [11,18], and it has been assessed that these Kupffer cells are constantly replenished by infiltrating monocytes [11,19], although this is contentious and some data contend that monocytes do not contribute to the resident pool [20–22]. Macrophages also stimulate hepatic progenitor cell activation in uninjured and injured liver with macrophage-derived TNF-related weak inducer of apoptosis (TWEAK) stimulating progenitor cell expansion via the FN14 receptor [6,23].

Cell therapy offers an alternative therapeutic avenue to increase the survival of patients with liver disease, for which there is no specific therapy. BM is considered a promising source of cells for liver regenerative therapy because the cells are readily obtained and manipulated [24]. During the past decade, a subset of clinical trials in cirrhotic patients has shown the safety, feasibility and improvement in liver function and regeneration after BM cell administration via multiple administration routes [24–30]. Although this has shown that in principle, phase 2 autologous stem cell trials can be conducted in cirrhotic patients, our preclinical work suggests that cultured and matured macrophages are likely to be more potent anti-fibrotic and pro-regenerative cells than unmanipulated hematopoietic stem cells [17]. Furthermore, if this cell therapy can be developed as an anti-fibrotic approach, then wider applications may be envisaged for conditions where macrophages have been shown to have a role in the resolution of tissue fibrosis or the promotion of tissue regeneration for example pulmonary [31], renal [32,33] and skin fibrosis [34].

Although a different, non-autologous macrophage product with differing indications and underlying aetiology, there are a subset of phase II trials utilizing macrophages with potential therapeutic benefit including the ongoing ONE study M-reg trial (clinicaltrials.gov, identifier NCT02085629) in which donor-derived regulatory macrophages (differentiated in the presence of interferon-γ) are intravenously infused to living-donor renal transplant recipients with the primary outcome measure being the incidence of biopsy confirmed acute rejection. Other phase II trials include a randomized controlled trial in which an injection of autologous incubated macrophages were given into the caudal boundary in acute, complete spinal cord injury [35], and an open label, multicenter, randomized trial in which patients with non-muscle invasive bladder cancer were given instillations of intravesical autologous macrophage cell therapy [36]. There has also been a phase II study in which patients with malignant pleural mesothelioma were given an intra-pleural infusion of activated macrophages [37]. The product in all these trials was well tolerated, although efficacy is uncertain.

The aim of this cohort study was to determine the feasibility of generating clinical-grade macrophages with a phenotype and function, which reflects previously characterized therapeutic macrophages in our model of cirrhotic disease with a view to macrophage therapy.

## Methods

### Methods for murine experiments

NOD CB17 Prkdc/^SCID^ mice were supplied by Charles River and housed in individually ventilated cages in a sterile animal facility with a 12-hour dark/light cycle and free access to food and water. All procedures were performed in accordance with UK Home Office guidelines (Animals [Scientific Procedures] Act 1986). Chronic liver fibrosis was induced in adult female mice over a 10-week period by twice weekly intraperitoneal injections of carbon tetrachloride (CCl_4_) dissolved in sterile olive oil at a concentration of 0.1 mL/kg for first 4 weeks increasing to 0.5 mL/kg for the remaining 6 weeks. One day after the 12th CCl_4_ injection (6 weeks), mice were randomly allocated to receive either cell (n = 8) or control (n = 7) medium injections via the spleen. The intra-splenic route was selected because it is anatomically easier than portal vein injection yet would still ensure maximal cell delivery compared to tail vein injection [38]. Cells were injected while mice were under isofluorane-induced anaesthesia. The macrophages used were isolated from an apheresis product from a healthy volunteer and matured for 7 days in culture in the presence of 100 ng/mL macrophage colony-stimulating factor (M-CSF). Macrophages were suspended in sterile saline at a density of 5 × 10^7^ cells/mL and 0.1 mL was injected via a 29-gauge needle. CCl_4_ administration continued for an additional 4 weeks. All mice were culled 4 weeks after the last macrophage/vehicle injection.

### Immunohistochemistry

Sirius red staining (BDH Laboratory Supplies) of formalin-fixed tissue sections was performed to identify collagen. Three-micrometer sections of formalin-fixed tissue were used for immunostaining of α-smooth muscle actin (α-SMA; Sigma) using a biotinylated anti-mouse secondary antibody (Vector) and F4/80 (Abcam) using a biotinylated ant-rat secondary antibody (Vector), then visualized using 3,3′-diaminobenzidine (Dako) then counterstained with Harris's hematoxylin.

### Assessment of tissue sections

Stained slides were blinded and a minimum of 20 serial, non-overlapping fields were photographed at ×100 magnification (Nikon Eclipse E600). For α-SMA, F480 and sirius red assessment, the percentage staining of the total field was measured using image analysis software (Adobe Photoshop, NIS elements).

### Quantification of messenger RNA levels by real-time reverse-transcription polymerase chain reaction

RNA was extracted from whole liver tissue using RNA extraction kits (Qiagen) according to the manufacturer's instructions. Complementary DNA was generated from 1 μg of RNA using the Superscript II kit (Invitrogen). Primers for TGF-β, COL1, MMP-9 and mouse TWEAK were purchased from Qiagen. A predesigned, validated eukaryotic 18S primer/probe set (Applied Biosystems) was used for internal control. Quantitative real-time polymerase chain reaction (qPCR) was performed using Express SYBR Green or TaqMan Express qPCR Supermix (Invitrogen). All reactions were performed in triplicate. Levels are expressed as dCT relative to glyceraldehyde 3-phosphate dehydrogenase.

### Liver function

Albumin, alanine transaminase (ALT), aspartate transaminase (AST), alkaline phosphatase (ALP) and bilirubin levels were measured in serum using commercial kits (albumin, bilirubin, ALT: Alpha Laboratories Ltd.; AST, ALP; Randox Laboratories) adapted for use on a Cobas Fara centrifugal analyser (Roche Diagnostics).

As these initial murine experiments demonstrated human monocyte-derived macrophages (HMDMs) decreased these markers of liver injury and fibrosis and increased markers of regeneration we then proceeded to determine the possibility of generating human clinical-grade macrophages with a phenotype and function that would reflect the therapeutic macrophages in our model of cirrhotic disease with a view to macrophage therapy.

### Methods for translational human study

Ethical approval was granted by the South East Scotland Research Ethics Committee 02, and use of buffy coats was covered by Scottish National Blood Transfusion Service (SNBTS) Sample Governance (13–12/14–02). Informed consent for apheresis donation was obtained in accordance with the Helsinki Declaration.

Initial phenotyping studies were performed on monocytes isolated from normal donor buffy coats. In addition, 10 healthy volunteer controls were recruited between August 2011 and March 2013 to demonstrate initial feasibility and then 11 patients with cirrhosis attending the Royal Infirmary of Edinburgh were recruited between January 2013 and October 2013.

Eligibility criteria included patients between 18 and 75 years with cirrhosis being defined by any one of the following: previous liver biopsy confirming histological features of cirrhosis, transient elastography (Fibroscan) >18 kPa and/or clinical and radiological features that in the opinion of the investigator correlated with a diagnosis of cirrhosis.

Exclusion criteria included refusal or inability to give informed consent to participate in the study, patients with viral hepatitis, average alcohol ingestion >21 units/week (male)/>14 units/week (female), ascites not well controlled with diuretic therapy in the preceding 3 months, encephalopathy requiring hospitalization for treatment in the past 3 months, portal hypertensive bleeding in the preceding 3 months, hepatocellular carcinoma, other cancer within the previous 5 years, previous liver transplant or currently on the waiting list, the presence of a clinically relevant acute illness that may compromise the patient's safe participation, and pregnancy and/or breastfeeding.

Patients initially attended for a “screening” visit to ensure trial eligibility and safety of participation. A full medical history, examination, vital signs, electrocardiograph and baseline blood tests to include a full blood count, urea and electrolytes, liver function tests and prothrombin time, were undertaken. Veins were also assessed to ensure suitability to undergo apheresis in which circulating monocytes would be peripherally extracted.

Healthy volunteers also attended a “screening” visit to ensure safety of participation as detailed above. In addition, 50 mL of peripheral blood was obtained from the healthy volunteers to ensure that the mononuclear cells would be comparable to those in the more favorable apheresis product in which it would be possible to obtain much higher yields.

Within 1 month from the initial screening visit, participants attended for apheresis.

### Isolation of CD14 monocytes from peripheral blood from healthy volunteers

Mononuclear cells were separated from whole blood obtained by venepuncture from healthy volunteers or from buffy coats and were separated by density centrifugation using Histopaque (Life Technologies Ltd). CD14 positive monocytes were isolated from the mononuclear cell fraction using CliniMACS CD14 reagent (to ensure compatibility with clinical-grade selections) and LS separation magnetic columns (Miltenyi Biotec). Briefly, cells were re-suspended to appropriate concentration in phosphate-buffered saline (PBS) plus ethylenediaminetetraacetic acid (2.5 mmol/L) and human serum albumin (PEA buffer), incubated with Clinimacs CD14 beads, then washed and passed through an LS column. After washing, the purified monocytes were eluted from the column, washed and re-suspended in medium for culture.

### Isolation of mononuclear cells from apheresis

A single procedure was performed using the Optia apheresis system and a sterile collection technique. A standard program for collection of mononuclear cells (MNCs) was employed. Between 2 and 2.5 blood volumes were processed where circumstances permitted. CD14 cells were isolated from the resultant product using CliniMACS CD14 reagent and an autoMACS separation apparatus (Miltenyi). Briefly, the apheresis product was diluted 1 in 5 with PBS, centrifuged then washed again with PBS. Cells were re-suspended in AutoMacs buffer (Miltenyi) and 0.5% fetal bovine solution (FBS) then counted and re-suspended to appropriate concentration and incubated with clinical grade CD14 microbeads. Cells were washed as before and re-suspended at manufacturer's recommended concentration for separation on autoMACS (Miltenyi). Cell suspension was passed through a pre-separation filter before being applied to the autoMACS and the number of separations performed was determined by the cell concentration as recommended by the manufacturer. The resultant monocytes were washed and re-suspended in medium for culture. The purity of the separation was assessed using a panel of antibodies against human leucocytes.

### Culture of CD14 monocytes

CD14+ve monocytes were cultured under low adhesion conditions in six-well plates (Corning) for buffy coat monocytes or MACS Good Manufacturing Practice (GMP) 100 mL expansion bags (Miltenyi Biotec) for all apheresis samples at a density of 2 × 10^6^ cells/mL in the presence of 100 ng/mL human recombinant M-CSF (Miltenyi) in a humidified atmosphere at 37°C (95%O_2_/5%CO_2_). Initial cultures were carried out in Iscove's Modified Eagle's Medium supplemented with 10% FBS from a validated GMP-compliant source (Life Technologies Ltd). Further experiments were carried out in serum-free AIMV medium (Invitrogen) to comply with GMP requirements. In all experiments a media change was performed on days 3 and 5. Low-adhesion plates were used because these best mimic the conditions in the expansion bags. Furthermore, if normal tissue culture plastic had been used, the macrophages would readily attach to the plastic, making it difficult to remove them without either mechanical disruption or enzymatic treatment, which could result in increased cell death and an altered macrophage phenotype.

### Flow cytometry

Cell surface expression of leucocyte markers in freshly isolated and day 7 matured cells was carried out by incubating cells with specific antibodies (final dilution 1:100). The following antibodies were used CD14-Alexa fluor 700 (M5E2; Biolegend) or PE (Miltenyi), CD16-Pacific blue (3G8; Biolegend), CD66b-FITC (80H3; AbD Serotech), CD209-perCPcy5.5 (9E9A8; Biolegend), CD163-PE (GHI/61; Biolegend), 25F9-eFluor 660 (eBiosciences), CD206-PE/Cy7 or FITC (15-2; Biolegend), CD11b perCPcy5.5 (ICRF44; Biolegend), CD93 APC (VIMD2; Biolegend), CD105 FITC (43A3; Biolegend), CD19-PE (HIB19; E-biosciences), CD45 Pacific-blue or Brilliant Violet 421(HI30; Biolegend, Miltenyi), HLA-DR alexa fluor 700 (L243; Biolegend), CCR2 (K036C2; Biolegend), and CD169-PE (7-239; Miltenyi). Cells were incubated on ice for 20 min, and then cells were washed in fluorescence-activated cell sorter buffer (PBS) containing 0.5% bovine serum albumin. Buffy coat monocytes and macrophages were gated to exclude debris, doublets and dead cells using DRAQ7 dead cell discriminator (Biolegend) and analyzed on a Canto II flow cytometer. Apheresis cells were fixed in 2% formalin before analysis on an LSRFortessa Flow cytometer (Beckton Dickenson). Data were analyzed using Flowjo software (Treestar).

Phagocytosis was determined by incubating human macrophages with pHrodo® Red *Staphylococcus aureus* Bioparticles (Invitrogen). Cells were incubated for 30 min at 37°C and then fixed in formalin, or for 1 hour then washed with ice-cold PBS-ethylenediaminetetraacetic acid and examined by fluorescence microscopy and flow cytometry.

### RNA extraction and quantitative reverse transcriptase-PCR

RNA was extracted from day 0 and day 7 CD14+ve healthy volunteer and cirrhotic cells for transcript analysis by qPCR. RNA was extracted from cells using RNA extraction kits (Qiagen) according to the manufacturer's instructions. Complementary DNA was generated from 1 μg of RNA using the Superscript II kit (Invitrogen). Predesigned, validated primer sets for MMP-9, CCL2, IL-10, CCL3, TNF, CXCL2 and TWEAK were purchased from Qiagen. A predesigned, validated eukaryotic 18S primer/probe set (Applied Biosystems) was used for internal control. qPCR was performed using Express SYBR Green or qPCR Supermix (Invitrogen). All reactions were performed in triplicate. Levels are expressed relative to 18S.

### Pathway array

A qPCR RT^2^ profiler array (PAHS-150ZG-4 human chemokine and cytokine array) was purchased from SABiosystems and was used as per the manufacturers protocol (n = 4 patients, n = 4 healthy volunteers at day 7).

### Statistical methods

Data values are presented as mean and standard error of the mean, SEM, or percentages unless otherwise stated. Statistical analysis was performed using SPSS (SPSS 19.0) and Graphpad Prism (GraphPad Software Inc.). Flow cytometric, yield and gene data between day 0 and day 7 was compared using Student's *t*-test. Data were routinely tested for normality using the Shapiro-Wilk test and for outliers using box plots. Results were considered statistically significant when *P* < 0.05.

## Results

### HMDMs improve murine liver fibrosis and function

**Figure 1:**
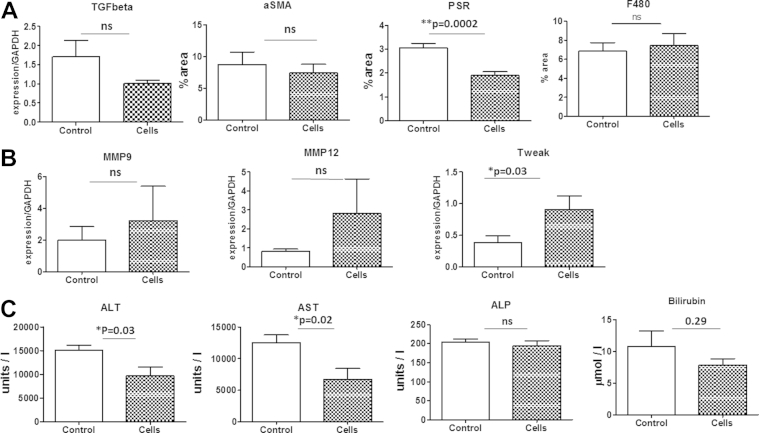
(A) Quantitation of collagen (picrosirius red staining [PSR]), myofibroblast activation (alpha-SMA) and macrophage recruitment (F480) in fibrotic mouse liver following HMDM injection, showing HMDMs decrease liver fibrosis, with no effect on macrophage recruitment. As measured quantitatively by PSR and alphaSMA and by gene expression/glyceraldehyde 3-phosphate dehydrogenase by TGF-β (B) HMDMs show down-regulation of TGF-β and up-regulation of MMP-12, MMP-9 and TWEAK and improvement in liver function tests (C) in NOD/SCID mice (n = 8) compared with control injection (n = 7; mean and SEM, ∗*P* < 0.05).

Our previous work has shown that infusions of mature mouse macrophages improve liver function and fibrosis in mice during CCl4-induced liver fibrosis [17] and stimulate liver regenerative pathways [6]. We therefore wanted to assess the effect of mature human macrophages on CCl4-induced liver fibrosis in mice. We used the NOD CB17 Prkdc^scid^ immunocompromised strain to test the efficacy of human macrophages. CD14+ monocytes were isolated from an apheresis product from a healthy volunteer and matured into macrophages for 7 days in the presence of M-CSF as described in Methods. Maturation was confirmed by expression of the mature macrophage marker 25F9 and the absence of the monocyte marker CD93 and of other leukocyte markers for neutrophils, T and B cells. On week 6 of a 10-week CCl_4_-induced injury protocol, 5 × 10^6^ cells were delivered intrasplenically. The livers of mice treated with human macrophages showed significantly reduced fibrosis as measured by Sirius red (*P* = 0.0002) and a trend to reduced myofibroblast activation as measured by alpha-SMA quantification (*P* = 0.59) and expression of the profibrotic gene TGF-β (*P* = 0.13; see Figure 1A).

The MMP family and TWEAK are critical regulatory components that result in the degradation of the extracellular matrix, key to the resolution of fibrosis [6]. Mice receiving HMDMs had non-significant trends to increased gene expression of MMP-12 (*P* = 0.31), MMP-9 (*P* = 0.61) and significantly increased TWEAK (*P* = 0.03). Mice receiving HMDMs had significant improvement in ALT (*P* = 0.03), AST (*P* = 0.02) and improvement in bilirubin (*P* = 0.29) in the treated group (Figure 1C). However, the same marked improvement was not seen in ALP.

**Figure 2:**
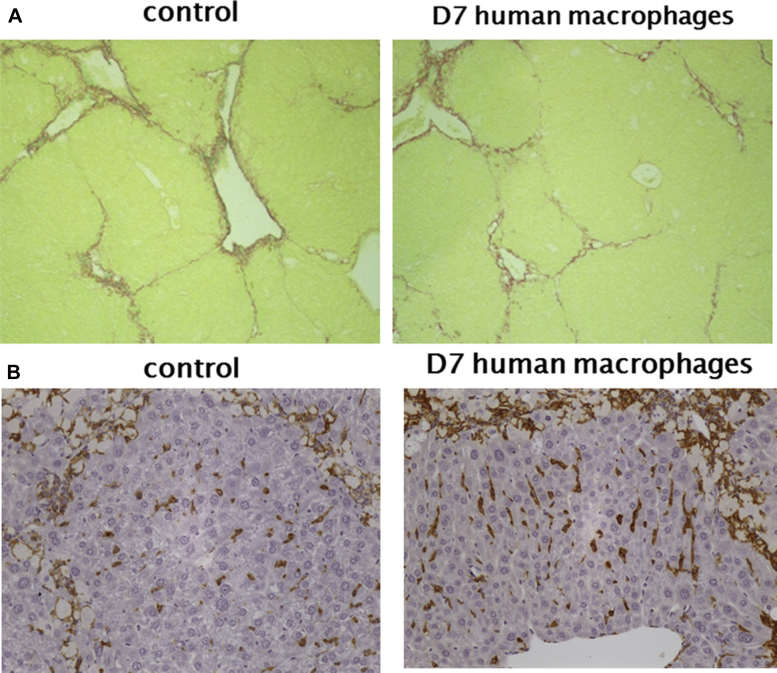
(A) Photomicrograph showing picrosirius red staining for hepatic collagens in CCl4-treated mice 4 weeks after injection with HMDMs or control medium, original magnification ×100. (B) Photomicrograph showing F480 staining 4 weeks after injection with HMDMs or control medium, original magnification ×200.

Although the reduction in fibrosis (the main clinical histological endpoint) is significantly improved by macrophage therapy, some inflammation-related indices such as MMP-9 and MMP-12 were not significantly changed. This may reflect the necessary use of immunocompromised mice and therefore lack of the amplified effect seen in previous studies, which were mouse-mouse studies [6,17] (Figure 1B). This amplified effect was mediated by recruitment of host macrophages and inflammatory cells, via injected macrophages chemokine secretion, to the scar areas in the immunocompetent mice and increased the effect of the single cell injection. There was no significant difference in the macrophage F480 staining 4 weeks after injection with either HMDMs or control medium (Figure 1A and 2).

### Baseline human data

A total of 10 healthy volunteers and 11 patients with cirrhosis underwent screening. All healthy volunteers completed the study. One patient failed screening as the patient's veins were not deemed suitable for apheresis, and one patient underwent apheresis, but it was not possible to separate the CD14+ cells sufficiently, most likely because the patient had a high granulocyte count. Thus nine patients completed the study. Because this was exploratory with a developing methodology, paired gene data between baseline and day 7 was obtained for cirrhotic patients and healthy volunteers but not statistically compared between the two groups.

Of the nine patients who completed the study, seven patients were men (77%), with a median age of 66 years (interquartile range [IQR] 63–69). Five patients had cirrhosis secondary to alcoholic liver disease (ALD; 55%), two secondary to both ALD and non-alcoholic fatty liver disease (NAFLD; 22%), one secondary to NAFLD (11%), and 1 cryptogenic (11%). At time of screening, all patients had Childs [39] grade A liver disease with a median Model for End-Stage Liver Disease [40] prognostic score of 8.3 (IQR 6.8–9.8). Of the seven patients in whom alcohol was an etiological factor, all patients had been abstinent for at least 6 months apart from one who drank eight units per day. There was no statistical difference between this patient and the others.

All patients tolerated apheresis well with no adverse events other than mild transient symptoms of hypocalcaemia (well recognized to occur with apheresis secondary to the citrate) quickly corrected with oral calcium. This was corroborated by a follow-up telephone call to the patient 1-month post-procedure.

With a median time of 9.5 months (IQR 4–14 months) since apheresis, no patients had experienced any liver-related complications. One patient had been diagnosed with type II diabetes mellitus and had a permanent pacemaker fitted. These events occurred 9 months after apheresis and were not assessed to be related.

### Baseline phenotypic characteristics of normal macrophages

**Figure 3:**
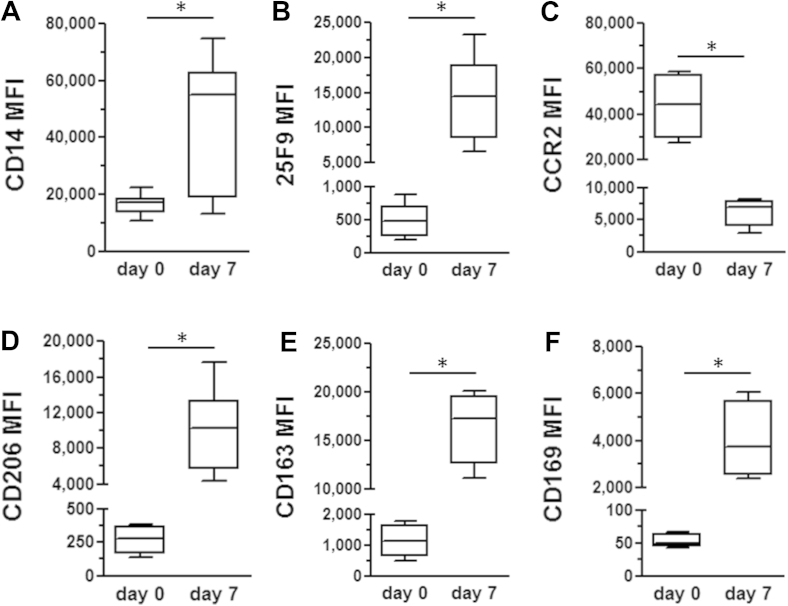
Quantitative analysis of macrophage phenotype in normal blood donors. Analysis of mean fluorescence index (MFI) of lineage (A and B), chemokine receptor (CCR) 2 (C) and phagocytosis markers (D–F) expressed on day 0 monocytes and day 7 after macrophage induction with M-CSF. All markers demonstrated significant increases (A, B, D–F) or decreases (C) in MFI at day 7. Data represent the means and SEM from 4 to 8 donors for each marker (∗*P* < 0.05).

Preliminary characterization of monocytes and macrophages from normal blood donors was performed using a panel of lineage and functional markers to determine changes between day 0 monocytes and day 7 macrophages. The mature macrophage marker 25F9 was significantly increased on day 7 macrophages, and there was a concomitant significant rise in markers associated with phagocytosis and tissue repair (CD163, CD169 and CD206) [41,42]. There was also a significant decrease in the level of the inflammatory cytokine receptor CCR2 (Figure 3). This phenotypic profile is indicative of disease resolution macrophages identified in the previous models of liver disease and in other pathologies [43,44]. This led us to determine whether this phenotype was consistent in macrophages generated from cirrhotic patients.

### Yields

**Supplementary Figure 1:**
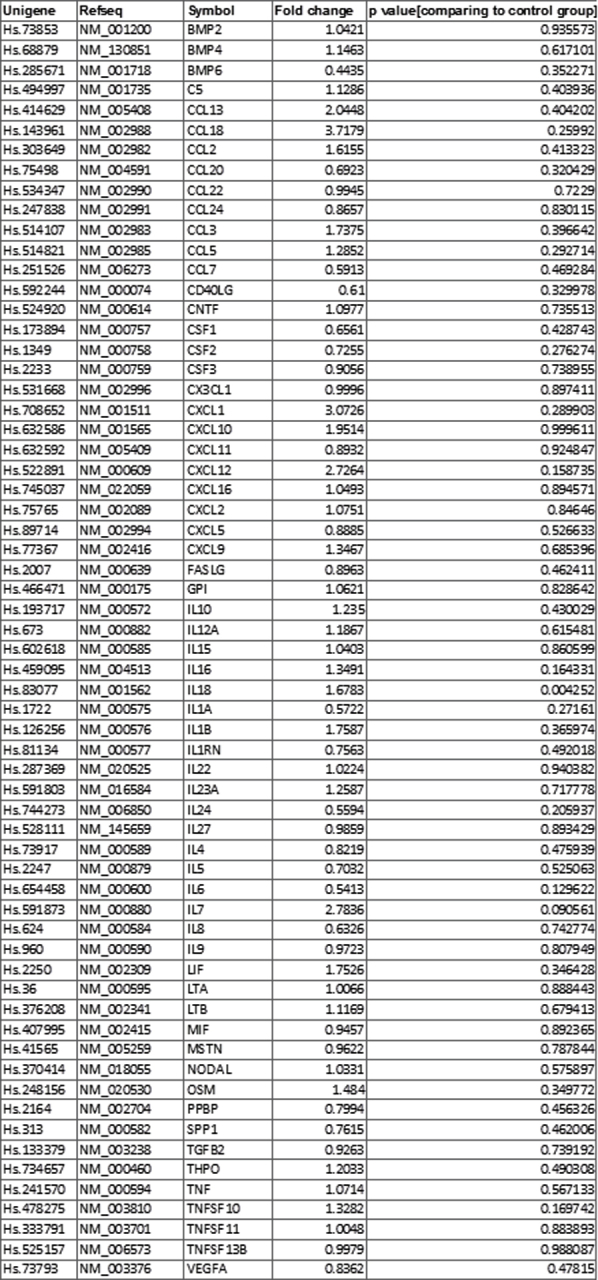
RT^2^ Profiler cytokine and chemokine PCR array of apheresis-derived macrophages from healthy volunteer and cirrhotic patients (n = 4 healthy volunteers, n = 4 cirrhotic patients).

The mean number of CD14+ monocytes isolated was 2.8 ± SEM 0.54 × 10^8^ from cirrhotic patients, n = 9 (18% ± SEM 5% CD14+ as a total of the MNCs processed) compared with 2.5 ± 0.6 × 10^8^, (8% ± 2% CD14+) for healthy volunteers, n = 10 (*t* = 0.38, *P* = 0.7, 95% confidence interval [CI] −1.4 to 2.0). There was also no significant difference in the mean yield of mature macrophages cultured from cirrhotic patients and healthy volunteers (0.9 × 10^8^ ± 0.4 × 10^8^ (48% ± 9% as a total of the MNCs processed), with more than 90% viability in cirrhotic patients and 0.65 × 10^8^ ± 0.16 × 10^8^ (47% ± 11%) for healthy volunteers (*t* = 0.43 with Welch's correction as unequal variances, *P* = 0.69, 95% CI −0.82 to 1.23). The day 7 macrophages had less than 5% apoptotic cells as measured by annexin V staining (Supplementary Figure 1).

The aim of the study was to validate a GMP-compliant protocol for the generation of macrophages from cirrhotic patients with a view to utilizing these cells as a cellular therapy. Therefore, we modified our protocol to incorporate a culture protocol that was animal-free and used a serum-free defined media (AIMV Invitrogen). There was no significant deterioration in yields between the AIMV media and serum-containing media (n = 2 healthy volunteers, 3.75 × 10^6^ CD14+ cultured, 37.5% AIMV versus 2.29 × 10^6^ CD14+ cultured FBS, 22.9% and 5.5 × 10^6^ CD14+ cultured, 18.3% AIMV versus 8.75 × 10^6^ CD14+ cultured, 29.2% FBS (*t* = 0.27, *P* = 0.81, 95% CI −13.5 to 15.29).

### Characterisation of macrophages from healthy volunteers and cirrhotics

**Figure 4:**
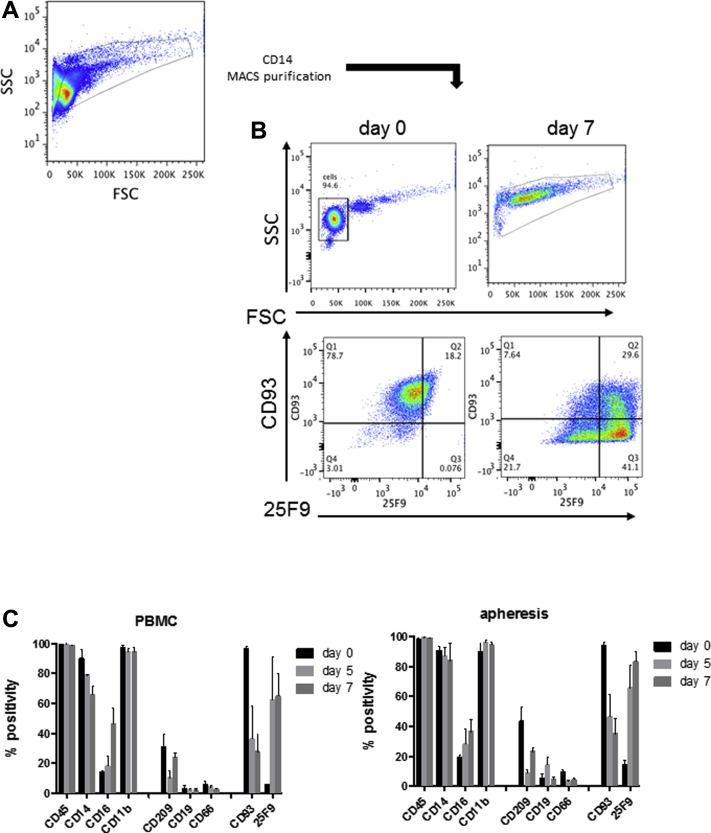
Characterization of monocyte-derived macrophages from apheresis from healthy volunteers. (A) Apheresis derived CD14 monocytes were isolated as described in Methods (n = 10) and cultured for 7 days in M-CSF-containing media with 10% fetal calf serum. (B) Forward/side scatter properties and CD93/25F9 expression in day 0 and day 7 matured cells. Marker expression is presented as % positivity compared with isotype controls. (C) Surface marker expression in CD14 monocytes isolated from peripheral blood (n = 5) or apheresis (n = 10) matured for 5 or 7 days with M-CSF. Where relevant, data are presented as mean and SEM.

Figure 4 shows the flow cytometry characterization of CD14+ monocytes and day 7 matured macrophages isolated from peripheral blood and apheresis from a healthy volunteer. Matured cells were larger and more granular than monocytes. Following 5–7 days in culture in low-adhesion expansion bags in the presence of M-CSF, the cells from both peripheral blood and apheresis had lost expression of the monocyte specific marker CD93 and gained expression of the mature macrophage marker 25F9 [45]. The cells exhibited coexpression of the pan-leucocyte marker CD45 and the myeloid marker CD11b with intermediate expression of CD16 and CD209 and low expression of the B-cell (CD19) and neutrophil (CD66b) markers. There was no significant difference between peripheral blood and apheresis-derived monocytes. Over 5 to 7 days in culture, cells increased expression of CD16 and 25F9 and reduced expression of the monocyte marker CD93.

**Figure 5:**
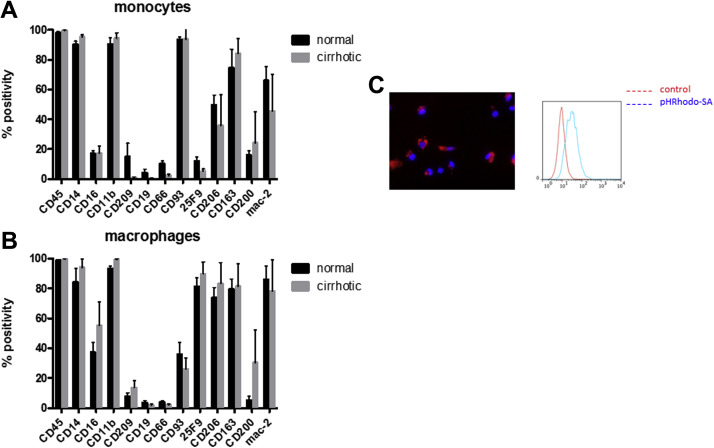
Characterization of monocytes and macrophages isolated from apheresis from healthy volunteer (n = 10) and cirrhotic patients (n = 9). (A) Surface marker expression in CD14 monocytes. (B) Surface marker expression relative to isotype control in day 7 matured macrophages. (C) Representative immunofluorescent image of mature control macrophages phagocytosing PhRhodamine-labeled *Staphylococcus aureus* (red) particles (red) nuclei are labelled with 4′,6-diamidino-2-phenylindole (blue). Where relevant, data are presented as mean and SEM.

The macrophages matured from the cirrhotic patients exhibited a similar cell surface marker profile as the healthy volunteers at day 0 and after 7 days in culture again with co-expression of CD45 and CD11b, intermediate expression of CD16 and CD209 (a dendritic cell marker but also a marker of the anti-inflammatory–like macrophage phenotype) and low expression of CD19 and CD66b (Figure 5). They also exhibited the same phagocytic features as would be seen with mature control macrophages (Figure 5C). All cells expressed differentiation markers consistent with an M2-like (anti-inflammatory) phenotype with high expression of CD206, CD163 and Mac-2.

**Figure 6:**
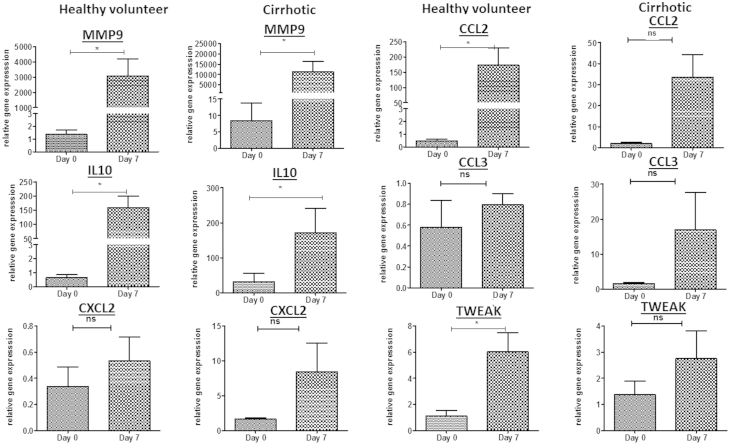
Gene change between day 0 apheresis derived monocytes and after 7 days of differentiation with AIMV media and 100 ng/mL M-CSF in healthy volunteers (n = 5) and cirrhotic patients (n = 8), (ns, nonsignificant, ∗*P* < 0.05, mean and SEM).

Transcript analysis from day 0 and day 7 cells showed that maturation resulted in an increase in expression of several MMPs and chemokines and cytokines. There were significant differences in MMP-9 (*P* = 0.04) and IL-10 (*P* = 0.04) in the genes in the cirrhotic patients between day 0 and day 7 and significant differences in MMP-9 (*P* = 0.03), CCL2 (*P* = 0.02), IL-10 (*P* = 0.006) and TWEAK (*P* = 0.02) in the controls (Figure 6). Up-regulation of TWEAK and IL-10 would be consistent with an M2 anti-inflammatory phenotype.

**Supplementary Figure 2:**
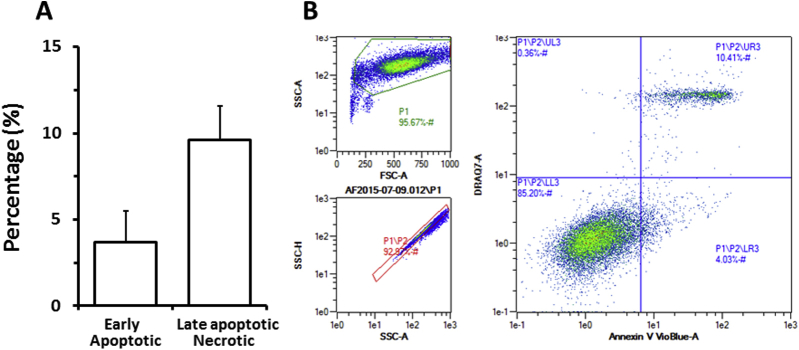
Apoptosis in macrophage cultures. (A) Early apoptosis (AnnV+) and late apoptosis/necrosis (AnnV+/DRAQ7+) measured in macrophage cultures from buffy coat CD14 cells at day 7 (mean plus SD, n = 4). (B) Representative plot of early apoptotic and late apoptotic/necrotic cells after excluding debris (P1) and doublet cells (P1/P2 gate).

**Figure 7:**
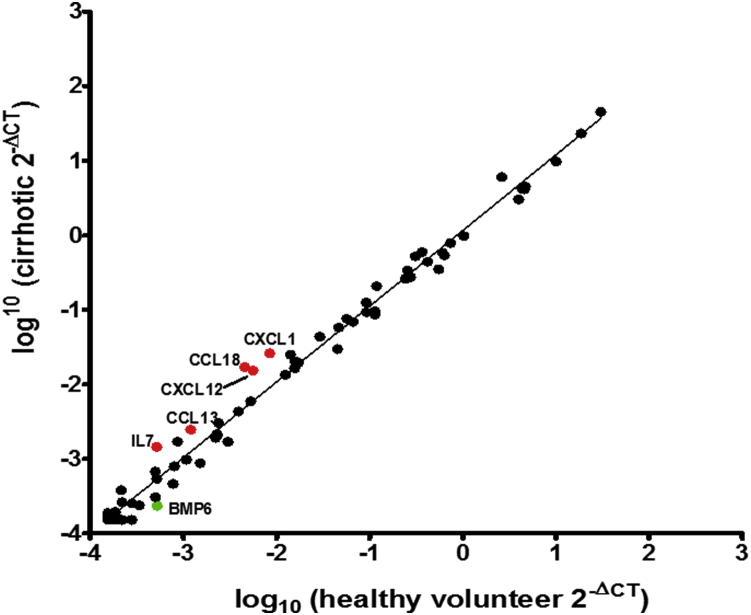
Scatter plot of RT^2^ Profiler cytokine and chemokine PCR array of apheresis-derived macrophages from healthy volunteers (n = 4) and cirrhotic patients (n = 4). Red circles show genes up-regulated ≥2-fold from cirrhotic patients relative to volunteers, green circles down-regulated ≥2-fold, all nonsignificant.

We also conducted an extensive human cytokine and chemokine array (Supplementary Figure 2) comparing the healthy volunteer and cirrhotic patients' macrophages at day 7. This revealed up-regulation from cirrhotic patients relative to HVs of IL-7, CCL17, CXCL12, CCL18 and CXCL and down-regulation of BMP6, but none were statistically significant (Figure 7).

## Discussion

We have shown for the first time that it is possible to differentiate CD14 positive monocytes from cirrhotic patients into macrophages in a GMP compliant environment, which demonstrate the same phenotypic profile as healthy controls. The macrophages show a significant increase in the mature macrophage marker 25F9; a significant rise in the phagocytosis and repair markers CD163, CD169 and CD206; and decrease in the inflammatory cytokine receptor CCR2.

Similar cell surface marker profiles were demonstrated, differentiation markers of the M2 (anti-inflammatory) phenotype were exhibited and yields were comparable between healthy volunteer and cirrhotic patients.

There was marked up-regulation in key cytokines in the fibrosis and regeneration pathways of the liver such as MMP-9 and IL-10, but the qPCR array comparing healthy volunteer and cirrhotic macrophages did not demonstrate a significant difference in the profiles. It is known that monocytes in cirrhotic patients exhibit different cytokine production patterns to healthy volunteers [15,16] but our data suggest that the monocyte-derived macrophages do not; this is important to establish before potential macrophage cellular therapy.

Although macrophages play a key contributory role in liver fibrosis by releasing pro-fibrogenic cytokines, promoting the survival of activated hepatic stellate cells (HSCs) [46] and promoting HSC migration through chemokines such as CCL2 [47], they also promote fibrosis resolution through enhanced extracellular matrix degradation via increased MMP expression [3]. “Pro-resolution” macrophages contribute to this resolution by contributing to the killing of HSCs—for example, through the expression of TNF-related apoptosis-inducing ligand [47].

Our earlier *in vivo* work, mouse BM-derived macrophages (and not undifferentiated monocytes) were infused in a murine model of liver fibrosis and resulted in improvement in clinically relevant parameters such as albumin and a reduction in fibrogenesis [17], demonstrating their therapeutic potential. This same therapeutic potential of HMDMs has been shown with our *in vivo* work presented here with significant improvements in the main histological endpoint: fibrosis and markers of liver function. The trends in MMP levels may have reached statistical significance if an intact recipient immune system were present. Previous studies in immunocompetent mice showed a significant cellular amplification mediated by injected cell-host cell recruitment to the hepatic scar areas [17]. Although a dose escalation study would be necessary, given 5 × 10^6^ seem to be therapeutic and well tolerated in a 20 g mouse, it could be expected that 1 × 10^9^ macrophages may be effective in humans, allowing for a safety margin (5 × 10^6^ 20 g mouse, 2.5 × 10^10^ for 100 kg, 1.25 × 10^10^ for 50 kg).

Importantly, we demonstrate for the first time the feasibility of differentiating cirrhotic patients' monocytes into functional macrophages comparable to healthy volunteers in a GMP environment. This gives hope that the *ex vivo* maturation of circulating monocytes can be a technique to produce therapeutic mature macrophages for clinical cell therapy. This paves the way for clinical studies in which these macrophages are re-infused into cirrhotic patients with the aim of improving liver function and fibrosis. This would represent, if successful, a potential significant advance in cell therapy for liver disease.

## References

[1] Tacke F., Zimmermann H.W. (2014). Macrophage heterogeneity in liver injury and fibrosis. J Hepatol.

[2] Tacke F. (2012). Functional role of intrahepatic monocyte subsets for the progression of liver inflammation and liver fibrosis in vivo. Fibrogenesis & tissue repair.

[3] Ramachandran P., Pellicoro A., Vernon M.A., Boulter L., Aucott R.L., Ali A. (2012). Differential Ly-6C expression identifies the recruited macrophage phenotype, which orchestrates the regression of murine liver fibrosis. Proc Nat Acad Sci U S A.

[4] Duffield J.S., Forbes S.J., Constandinou C.M., Clay S., Partolina M., Vuthoori S. (2005). Selective depletion of macrophages reveals distinct, opposing roles during liver injury and repair. J Clin Invest.

[5] Forbes S.J., Rosenthal N. (2014). Preparing the ground for tissue regeneration: from mechanism to therapy. Nat Med.

[6] Bird T.G., Lu W.Y., Boulter L., Gordon-Keylock S., Ridgway R.A., Williams M.J. (2013). Bone marrow injection stimulates hepatic ductular reactions in the absence of injury via macrophage-mediated TWEAK signaling. Proc Nat Acad Sci U S A.

[7] Jenkins S.J., Ruckerl D., Cook P.C., Jones L.H., Finkelman F.D., van Rooijen N. (2011). Local macrophage proliferation, rather than recruitment from the blood, is a signature of TH2 inflammation. Science.

[8] Murray P.J., Wynn T.A. (2011). Protective and pathogenic functions of macrophage subsets. Nat Rev Immunol.

[9] Mosser D.M., Edwards J.P. (2008). Exploring the full spectrum of macrophage activation. Nat Rev Immunol.

[10] Murray P.J., Allen J.E., Biswas S.K., Fisher E.A., Gilroy D.W., Goerdt S. (2014). Macrophage activation and polarization: nomenclature and experimental guidelines. Immunity.

[11] Karlmark K.R., Weiskirchen R., Zimmermann H.W., Gassler N., Ginhoux F., Weber C. (2009). Hepatic recruitment of the inflammatory Gr1+ monocyte subset upon liver injury promotes hepatic fibrosis. Hepatology.

[12] Wynn T.A., Barron L. (2010). Macrophages: master regulators of inflammation and fibrosis. Semin Liver Dis.

[13] Fallowfield J.A., Mizuno M., Kendall T.J., Constandinou C.M., Benyon R.C., Duffield J.S. (2007). Scar-associated macrophages are a major source of hepatic matrix metalloproteinase-13 and facilitate the resolution of murine hepatic fibrosis. Journal of immunology.

[14] Popov Y., Sverdlov D.Y., Bhaskar K.R., Sharma A.K., Millonig G., Patsenker E. (2010). Macrophage-mediated phagocytosis of apoptotic cholangiocytes contributes to reversal of experimental biliary fibrosis. Am J Physiol Gastrointest Liver physiol.

[15] Zimmermann H.W., Seidler S., Nattermann J., Gassler N., Hellerbrand C., Zernecke A. (2010). Functional contribution of elevated circulating and hepatic non-classical CD14CD16 monocytes to inflammation and human liver fibrosis. PloS one.

[16] Peter J., Frey O., Stallmach A., Bruns T. (2013). Attenuated antigen-specific T cell responses in cirrhosis are accompanied by elevated serum interleukin-10 levels and down-regulation of HLA-DR on monocytes. BMC Gastroenterol.

[17] Thomas J.A., Pope C., Wojtacha D., Robson A.J., Gordon-Walker T.T., Hartland S. (2011). Macrophage therapy for murine liver fibrosis recruits host effector cells improving fibrosis, regeneration and function. Hepatology.

[18] Heymann F., Trautwein C., Tacke F. (2009). Monocytes and macrophages as cellular targets in liver fibrosis. Inflamm Allergy Drug Targets.

[19] Klein I., Cornejo J.C., Polakos N.K., John B., Wuensch S.A., Topham D.J. (2007). Kupffer cell heterogeneity: functional properties of bone marrow derived and sessile hepatic macrophages. Blood.

[20] Yamamoto T., Naito M., Moriyama H., Umezu H., Matsuo H., Kiwada H. (1996). Repopulation of murine Kupffer cells after intravenous administration of liposome-encapsulated dichloromethylene diphosphonate. Am J Pathol.

[21] Naito M., Umeda S., Yamamoto T., Moriyama H., Umezu H., Hasegawa G. (1996). Development, differentiation, and phenotypic heterogeneity of murine tissue macrophages. J Leukoc Biol.

[22] Shepard J.L., Zon L.I. (2000). Developmental derivation of embryonic and adult macrophages. Cur Opin Hematol.

[23] Boulter L., Govaere O., Bird T.G., Radulescu S., Ramachandran P., Pellicoro A. (2012). Macrophage-derived Wnt opposes Notch signaling to specify hepatic progenitor cell fate in chronic liver disease. Nat Med.

[24] Houlihan D.D., Newsome P.N. (2008). Critical review of clinical trials of bone marrow stem cells in liver disease. Gastroenterology.

[25] Takami T., Terai S., Sakaida I. (2011). Novel findings for the development of drug therapy for various liver diseases: Current state and future prospects for our liver regeneration therapy using autologous bone marrow cells for decompensated liver cirrhosis patients. J Pharmacol Sci.

[26] am Esch J.S., Knoefel W.T., Klein M., Ghodsizad A., Fuerst G., Poll L.W. (2005). Portal application of autologous CD133+ bone marrow cells to the liver: a novel concept to support hepatic regeneration. Stem cells.

[27] Levicar N., Pai M., Habib N.A., Tait P., Jiao L.R., Marley S.B. (2008). Long-term clinical results of autologous infusion of mobilized adult bone marrow derived CD34+ cells in patients with chronic liver disease. Cell Prolif.

[28] Lyra A.C., Soares M.B., da Silva L.F., Braga E.L., Oliveira S.A., Fortes M.F. (2010). Infusion of autologous bone marrow mononuclear cells through hepatic artery results in a short-term improvement of liver function in patients with chronic liver disease: a pilot randomized controlled study. Eur J Gastroenterol Hepatol.

[29] Nikeghbalian S., Pournasr B., Aghdami N., Rasekhi A., Geramizadeh B., Hosseini Asl S.M. (2011). Autologous transplantation of bone marrow-derived mononuclear and CD133(+) cells in patients with decompensated cirrhosis. Arch Iran Med.

[30] Moore J.K., Stutchfield B.M., Forbes S.J. (2014). Systematic review: the effects of autologous stem cell therapy for patients with liver disease. Ali Pharmacol Ther.

[31] Gibbons M.A., MacKinnon A.C., Ramachandran P., Dhaliwal K., Duffin R., Phythian-Adams A.T. (2011). Ly6Chi monocytes direct alternatively activated profibrotic macrophage regulation of lung fibrosis. Am J Respir Crit Care Med.

[32] Lee S., Huen S., Nishio H., Nishio S., Lee H.K., Choi B.S. (2011). Distinct macrophage phenotypes contribute to kidney injury and repair. J Am Soc Nephrol.

[33] Duffield J.S. (2010). Macrophages and immunologic inflammation of the kidney. Semin Nephrol.

[34] Sindrilaru A., Scharffetter-Kochanek K. (2013). Disclosure of the Culprits: Macrophages-Versatile Regulators of Wound Healing. Adv Wound Care.

[35] Lammertse D.P., Jones L.A., Charlifue S.B., Kirshblum S.C., Apple D.F., Ragnarsson K.T. (2012). Autologous incubated macrophage therapy in acute, complete spinal cord injury: results of the phase 2 randomized controlled multicenter trial. Spinal cord.

[36] Burger M., Thiounn N., Denzinger S., Kondas J., Benoit G., Chapado M.S. (2010). The application of adjuvant autologous antravesical macrophage cell therapy vs. BCG in non-muscle invasive bladder cancer: a multicenter, randomized trial. Journal of translational medicine.

[37] Monnet I., Breau J.L., Moro D., Lena H., Eymard J.C., Menard O. (2002). Intrapleural infusion of activated macrophages and gamma-interferon in malignant pleural mesothelioma: a phase II study. Chest.

[38] Goto Y., Ohashi K., Utoh R., Yamamoto M., Okano T. (2011). Hepatocyte transplantation through the hepatic vein: a new route of cell transplantation to the liver. Cell Transplant.

[39] Pugh R.N., Murray-Lyon I.M., Dawson J.L., Pietroni M.C., Williams R. (1973). Transection of the oesophagus for bleeding oesophageal varices. Br J Surg.

[40] Malinchoc M., Kamath P.S., Gordon F.D., Peine C.J., Rank J., ter Borg P.C. (2000). A model to predict poor survival in patients undergoing transjugular intrahepatic portosystemic shunts. Hepatology.

[41] Wang H., Melton D.W., Porter L., Sarwar Z.U., McManus L.M., Shireman P.K. (2014). Altered macrophage phenotype transition impairs skeletal muscle regeneration. Am J Pathol.

[42] Bourlier V., Zakaroff-Girard A., Miranville A., De Barros S., Maumus M., Sengenes C. (2008). Remodeling phenotype of human subcutaneous adipose tissue macrophages. Circulation.

[43] Chow A., Huggins M., Ahmed J., Hashimoto D., Lucas D., Kunisaki Y. (2013). CD169(+) macrophages provide a niche promoting erythropoiesis under homeostasis and stress. Nat Med.

[44] Novak M.L., Koh T.J. (2013). Macrophage phenotypes during tissue repair. J Leukoc Biol.

[45] Pilling D., Fan T., Huang D., Kaul B., Gomer R.H. (2009). Identification of markers that distinguish monocyte-derived fibrocytes from monocytes, macrophages, and fibroblasts. PloS one.

[46] Pradere J.P., Kluwe J., De Minicis S., Jiao J.J., Gwak G.Y., Dapito D.H. (2013). Hepatic macrophages but not dendritic cells contribute to liver fibrosis by promoting the survival of activated hepatic stellate cells in mice. Hepatology.

[47] Pellicoro A., Ramachandran P., Iredale J.P., Fallowfield J.A. (2014). Liver fibrosis and repair: immune regulation of wound healing in a solid organ. Nat Rev Immunol.

